# Transcriptomic Characteristics Associated With Aging in the Thyroid Gland

**DOI:** 10.3389/fnut.2022.859702

**Published:** 2022-05-25

**Authors:** Chien-Liang Liu, Ming-Nan Chien, Yi-Chiung Hsu, Shih-Ping Cheng

**Affiliations:** ^1^Department of Surgery, MacKay Memorial Hospital and Mackay Medical College, Taipei, Taiwan; ^2^Division of Endocrinology and Metabolism, Department of Internal Medicine, MacKay Memorial Hospital and Mackay Medical College, Taipei, Taiwan; ^3^Department of Biomedical Sciences and Engineering, National Central University, Taoyuan City, Taiwan; ^4^Institute of Biomedical Sciences, Mackay Medical College, New Taipei City, Taiwan; ^5^Department of Pharmacology, School of Medicine, College of Medicine, Taipei Medical University, Taipei, Taiwan

**Keywords:** thyroid gland, aging, immune response, mitochondria, cytoskeletal proteins

## Abstract

The aging thyroid is associated with a plethora of morphological and functional changes. Limited studies have addressed the gene expression signature in the aging thyroid, except for sporadic reports using data from postmortem samples in the Genotype-Tissue Expression (GTEx) project. In this investigation, we analyzed the RNA sequencing data of 58 samples of normal-appearing counterpart thyroid tissues from The Cancer Genome Atlas. Aging-correlated genes were identified by determining the Spearman rank-order correlation between patient age and gene expression level. Additionally, we performed gene set enrichment analysis and conducted a weighted correlation network analysis. The results were compared with those analyzed using the GTEx data. The over-represented protein class of aging-correlated genes is mainly metabolite interconversion enzymes. Our analyses identified alterations in immune and inflammatory responses, mitochondrial functions, cytoskeletal proteins, as well as amino acid and cytochrome P450 metabolism. There was no significant association between thyroid differentiation and age. Our findings may shed molecular light on thyroid disorders in the geriatric population.

## Introduction

The intricate relationship between endocrine systems and aging has long been recognized. The thyroid gland is interconnected with the molecular pillars of aging: metabolism, inflammation, epigenetics, adaptation to stress, stem cell renewal, proteostasis, and macromolecular damage (1). Concentrations of thyroid-stimulating hormone (TSH) and parathyroid hormone appear to increase with age (2). Thyroid dysfunction affects the cardiovascular system and cognitive function, while disturbance in calcium and bone homeostasis from parathyroid dysfunction increases the risk of falls and fractures. Recently, we found that age plays an important role in the response of B-type natriuretic peptides to parathyroidectomies (3).

We need to investigate the adaptive dynamics associated with aging at the molecular scale to dissect the physiological alterations in the endocrine system. Using transcriptome profiles from postmortem samples in the Genotype-Tissue Expression (GTEx) project, a previous analysis highlighted that age-related gene expression change was tissue-specific although cross-tissue synchronization was observed in some essential tissues such as the heart and lungs (4). Interestingly, the thyroid gland exhibited the smallest number of age-associated genes among the nine human tissues examined. A subsequent study indicated downregulation of genes related to mitochondrial and proteasomal functions in the aging thyroid (5). Nonetheless, RNA degradation caused by cold ischemia after death can potentially lead to biases in differential expression analysis. It is recognized that gene expression from the GTEx project is influenced by postmortem intervals (6).

To overcome this obstacle, in this study, we systematically determined the characteristics of age-associated gene expression changes from RNA-seq samples from The Cancer Genome Atlas (TCGA). Our study may contribute to a better understanding of the process of thyroid aging at the transcriptional level.

## Materials and Methods

### Data Acquisition

RNA sequencing data from the TCGA papillary thyroid cancer (THCA) database were obtained from the Genomic Data Common data portal (https://portal.gdc.cancer.gov/). As per the TCGA processing pipeline, biospecimens were collected from patients who received no prior treatment and underwent surgical resection for papillary thyroid cancer. Snap-frozen samples were analyzed at a centralized facility. Gene expression data from Illumina HiSeq RNA sequencing were acquired as RNA-Seq by expectation-maximization (RSEM) values (7). Aside from RNA sequencing data from the tumor sample of 505 patients, TCGA contained transcriptome data on normal-appearing adjacent thyroid tissue from 59 patients. One patient (TCGA-EL-A3H2) who had a history of neck radiation exposure was excluded from the analysis. In total, transcriptome profiling from 58 TCGA subjects was analyzed. Age at the time of surgery was available for all patients.

GTEx data (v8 release) was obtained from the GTEx portal (https://gtexportal.org/home/datasets). Age groups (20–29, 30–39, 40–49, 50–59, 60–69, and 70–79 years) but no exact age was available. RNA was extracted from multiple tissues of postmortem donors. For the thyroid gland, 653 transcriptome data were obtained for grossly non-nodular normal regions from either side (whichever side was observed to be more normal). We excluded 49 samples in which thyroiditis was present to eliminate potential effects of autoimmune activity (5). Eventually, RNA-seq profiles from 604 GTEx subjects were included for analysis.

### Aging-Correlated Genes

For each gene, a Spearman rank-order correlation between the expression level and patient age was calculated. The false discovery rate (FDR) was estimated for each gene through a method developed by Benjamini and Hochberg to control for false positives (8). Aging-correlated genes were arbitrarily defined as genes with a Benjamini-Hochberg FDR <0.1.

### Pathway Analysis

The aging-correlated genes were analyzed using the protein classification tool of Protein ANalysis THrough Evolutionary Relationships (PANTHER), version 17.0 (9). The Kyoto Encyclopedia of Genes and Genomes (KEGG), Reactome, and BioCarta pathways were used for the functional annotation, and over-represented pathways were considered significant at 0.05 after Benjamini-Hochberg correction for multiple tests.

### Gene Set Enrichment Analysis

To validate the relationship of patient age with thyroid transcriptome, we divided the TCGA cohort into two groups with a cutoff of 60 years: young (*n* = 43) and old (*n* = 15) groups. Functional enrichment analyses were performed using GSEA (1,000 permutations) with referenced data sets from the hallmark gene sets (C1) as well as KEGG and Reactome subsets of canonical pathways (C2) (10). Positive and negative enrichment were separated to illustrate the forward and backward linkages. For the GTEx cohort, transcriptome profiling was compared between the young (20–59 years, *n* = 387) and old (60–79 years, *n* = 217) groups.

### Weighted Correlation Network Analysis

**Figure 1:**
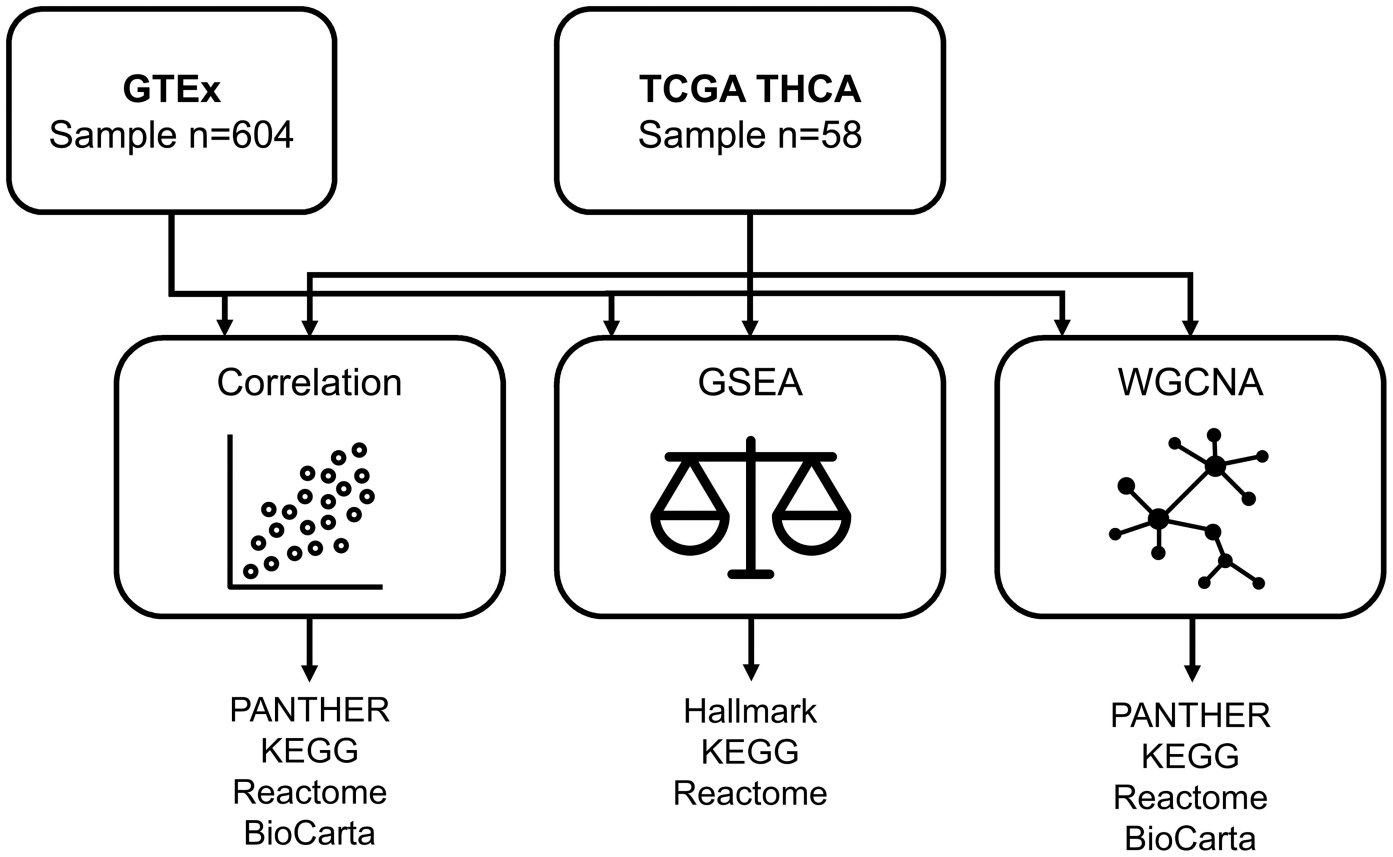
Study flowchart.

We used the WGCNA approach to identify co-expression modules based on pairwise Pearson correlations (11). Modules with significant correlations with age were further subjected to pathway enrichment analyses including the PANTHER protein class, KEGG, Reactome, and BioCarta (Figure 1).

### Thyroid Differentiation

The initial TCGA analysis developed a thyroid differentiation score through clustering of thyroid metabolism and function genes (12). The mRNA expression levels of 16 genes (DIO1, DIO2, DUOX1, DUOX2, FOXE1, GLIS3, NKX2-1, PAX8, SLC26A4, SLC5A5, SLC5A8, TG, THRA, THRB, TPO, and TSHR) were used to derive the thyroid differentiation score. In the present study, RSEM values of the 16 thyroid differentiation genes were log-transformed and median centered. A thyroid differentiation index was calculated by summing up the values across 16 genes for each sample.

## Results

### Aging-Correlated Genes

**Figure 2:**
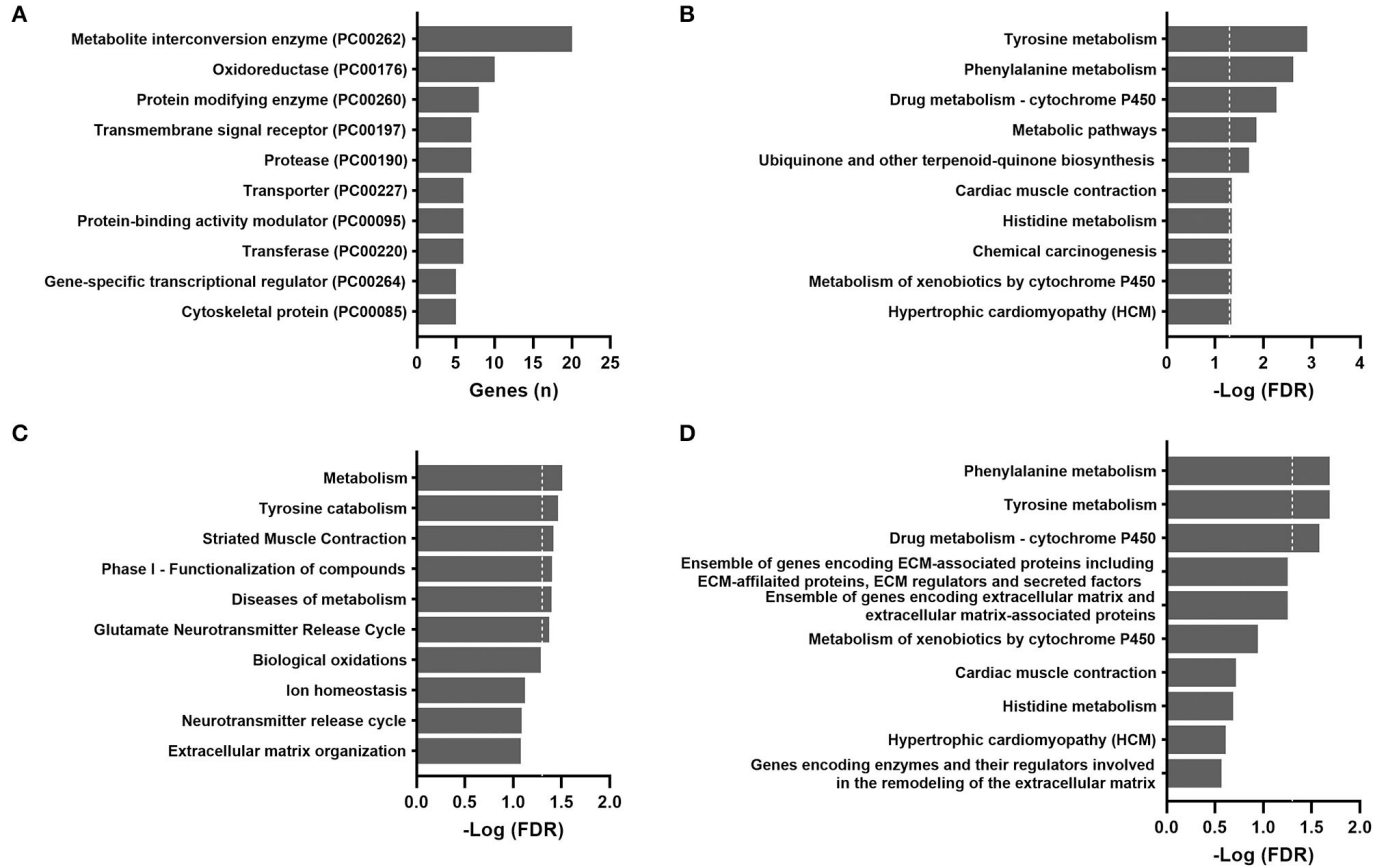
Over-representation pathway analysis of aging-correlated genes in the TCGA THCA dataset: **(A)** PANTHER protein class, **(B)** KEGG, **(C)** Reactome, and **(D)** BioCarta. The top 10 classes or pathways are listed. Values above the dashed line represent significant enrichment (5% level of significance). FDR, false discovery rate.

The 58 TCGA subjects included 42 (72%) women and 16 (28%) men. The median age was 42 years. Among the 43 subjects with race/ethnicity reported, approximately 72% were white, 12% were black, 9% were Hispanic, and 7% were Asian. The association between patient age and gene expression was tested by Spearman's rank-order correlation, and 147 genes were considered to be aging-correlated with an FDR <0.1 after multiple testing corrections. The complete list of these correlation coefficients is reported in Supplementary Table 1. The protein class most over-represented in aging-correlated genes was metabolite interconversion enzymes according to the PANTHER protein classification (Figure 2A).

**Figure 3:**
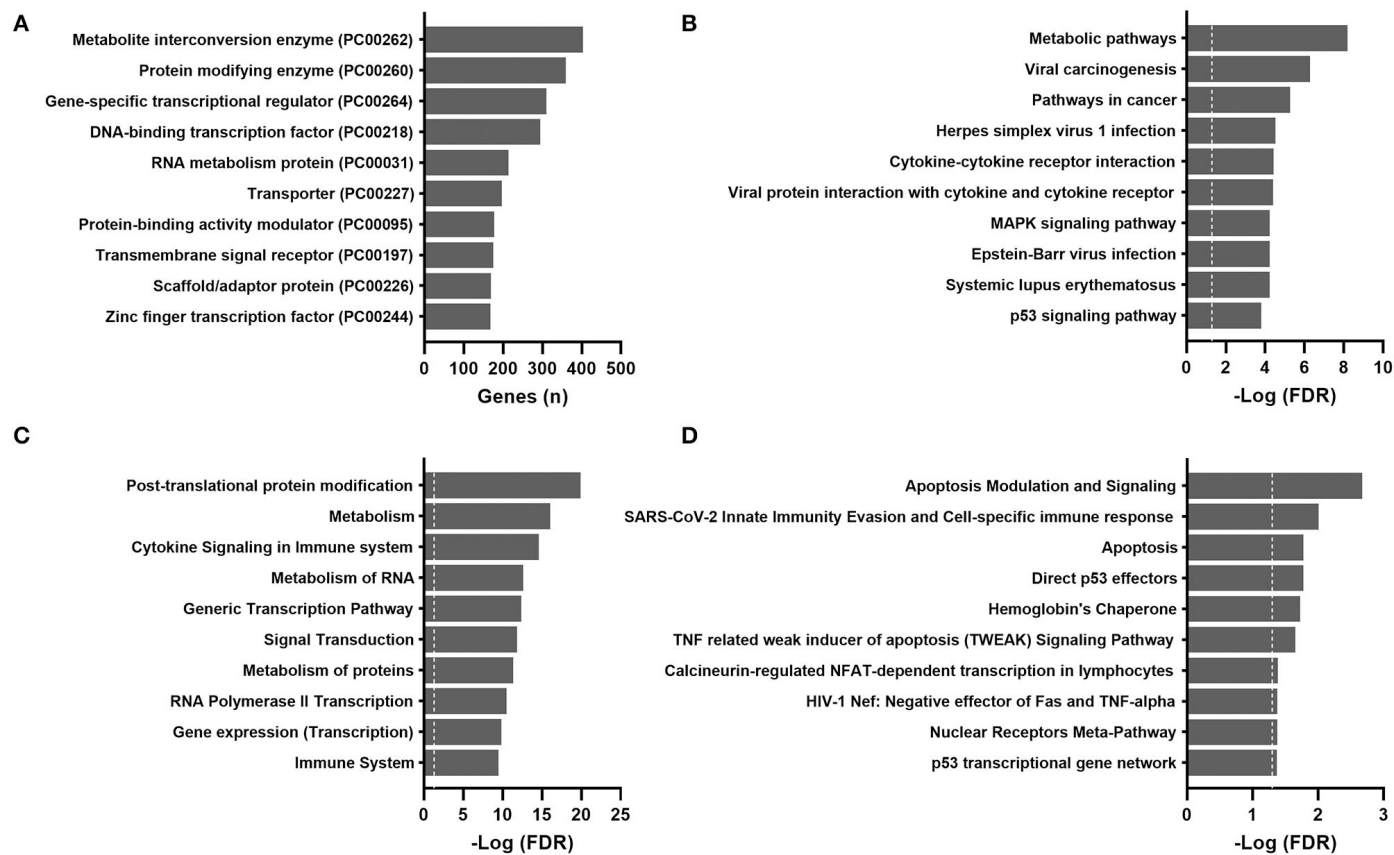
Over-representation pathway analysis of aging-correlated genes in the GTEx dataset: **(A)** PANTHER protein class, **(B)** KEGG, **(C)** Reactome, and **(D)** BioCarta. The top 10 classes or pathways are listed. Values above the dashed line represent significant enrichment (5% level of significance). FDR, false discovery rate.

For validation purposes, Spearman's rank-order correlations were calculated for 604 GTEx samples (Supplementary Table 2). The most over-represented protein class was also metabolite interconversion enzymes (Figure 3A). A total of 72 aging-correlated genes were common in both TCGA and GTEx datasets. The top 10 correlated genes are listed in Table 1.

**Table 1:** Top 10 aging-correlated genes in the thyroid gland.

**Gene**	**Description**	**TCGA THCA**		**GTEx**	
		**Rho**	**FDR**	**Rho**	**FDR**
PTCHD4	Patched domain containing 4	0.5424	0.0180	0.4718	4.54E-30
SPATA18	Spermatogenesis associated 18	0.4978	0.0607	0.3932	1.66E-19
POU3F4	POU class 3 homeobox 4	0.6037	0.0031	0.3197	1.09E-12
BMPR1B	Bone morphogenetic protein receptor type 1B	0.6019	0.0031	0.2919	1.25E-10
EYA4	EYA transcriptional coactivator and phosphatase 4	0.5530	0.0137	0.3081	8.39E-12
RPS27L	Ribosomal protein S27 like	0.5048	0.0501	0.2885	2.19E-10
DAPL1	Death associated protein like 1	0.5550	0.0130	0.2626	1.00E-08
SMCO3	Single-pass membrane protein with coiled-coil domains 3	0.5567	0.0136	0.2525	4.09E-08
TAFA3	TAFA chemokine like family member 3	0.5325	0.0234	0.2604	1.38E-08
NSMF	NMDA receptor synaptonuclear signaling and neuronal migration factor	0.4680	0.0907	0.2800	7.53E-10

*FDR, Benjamini-Hochberg false discovery rate; Rho, Spearman rank-order correlation coefficient*.

### Over-Representation Pathway Analysis

For the TCGA cohort, the most significantly enriched pathways identified by the KEGG, Reactome, and BioCarta were metabolic pathways, particularly amino acid metabolism (tyrosine, phenylalanine, and histidine) and cytochrome P450 metabolism (Figure 2). Actin and other cytoskeletal proteins correlated with aging were also enriched in pathways involved in cardiac or striated muscle contraction (KEGG and Reactome) or hypertrophic cardiomyopathy (KEGG).

The results of pathway analyses for the 604 GTEx samples are shown in Figure 3. Consistently, metabolic pathways (metabolism of RNA and proteins) remained highly ranked in the KEGG and Reactome. Pathways related to infection and immune responses were enriched in all databases, including cytokine signaling and cytokine-receptor interactions. Apoptosis and p53 pathways were enriched in the GTEx but not the TCGA samples.

### GSEA and WGCNA Approaches in the TCGA Cohort

**Figure 4:**
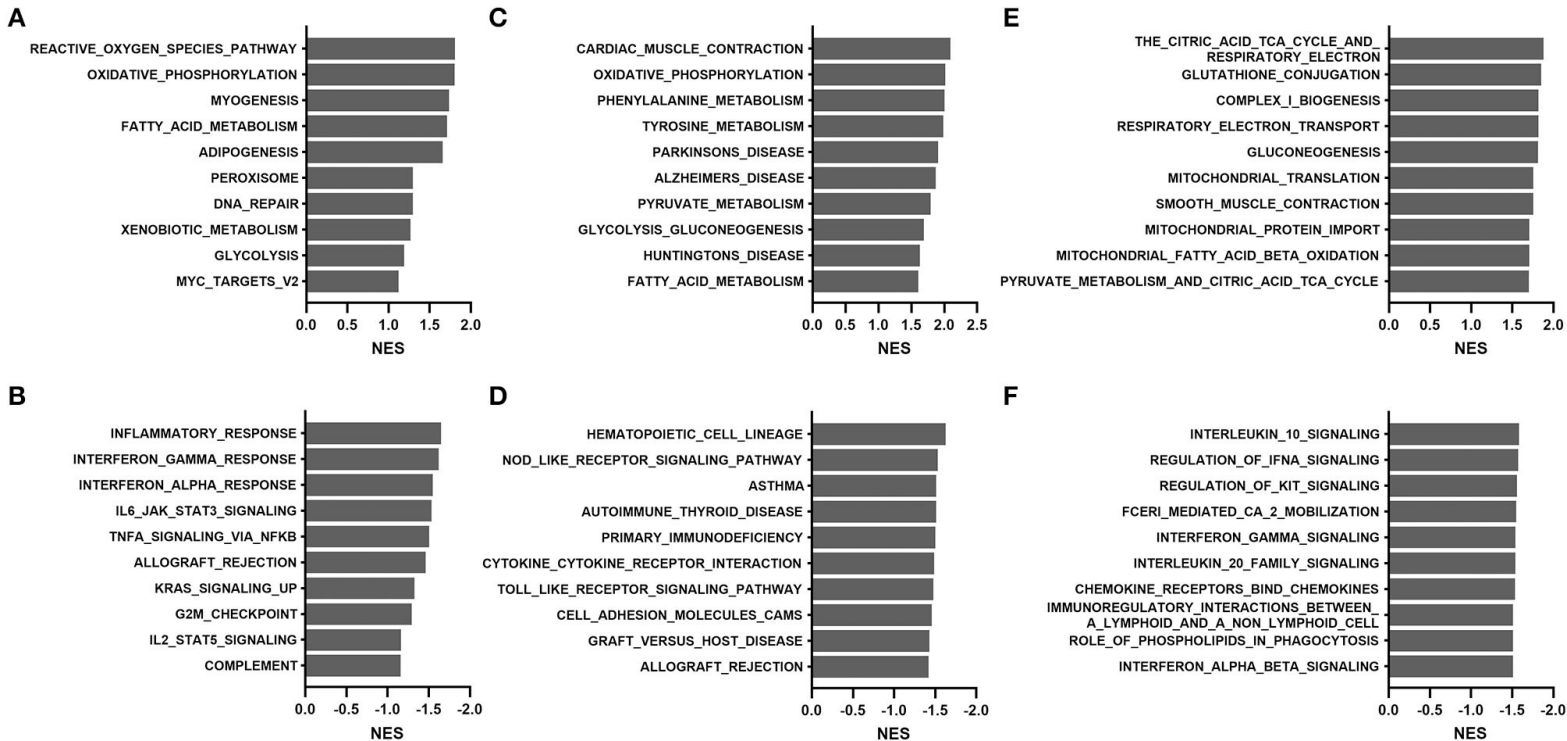
Gene set enrichment analysis of the TCGA THCA dataset comparing the young (15–58 years) and old (60–81 years) groups: **(A,B)** hallmark, **(C,D)** KEGG, and **(E,F)** Reactome. **(A,C,E)**, positive enrichment; **(B,D,F)**, negative enrichment. The top 10 hallmarks or pathways are listed. NES, normalized enrichment score.

To overcome the drawbacks of the over-representation approach in which the results depend on the cutoff used and the remaining genes not in the list are ignored, we performed GSEA by comparing transcriptomes between the young (median age, 36 years; range, 15 to 58) and old (median age, 69 years; range, 60 to 81) groups. As shown in Figure 4, the most positively enriched pathways were metabolic pathways, including oxidative phosphorylation, tyrosine metabolism, and fatty acid metabolism. Additionally, pathways related to mitochondrial function and muscle contraction were consistently enriched across databases. Most negative enrichment pathways involve inflammatory or defense responses, particularly interferon signaling and pro-/anti-inflammatory cytokine signaling. These findings were consistent with results of the over-representation analysis in GTEx samples.

**Figure 5:**
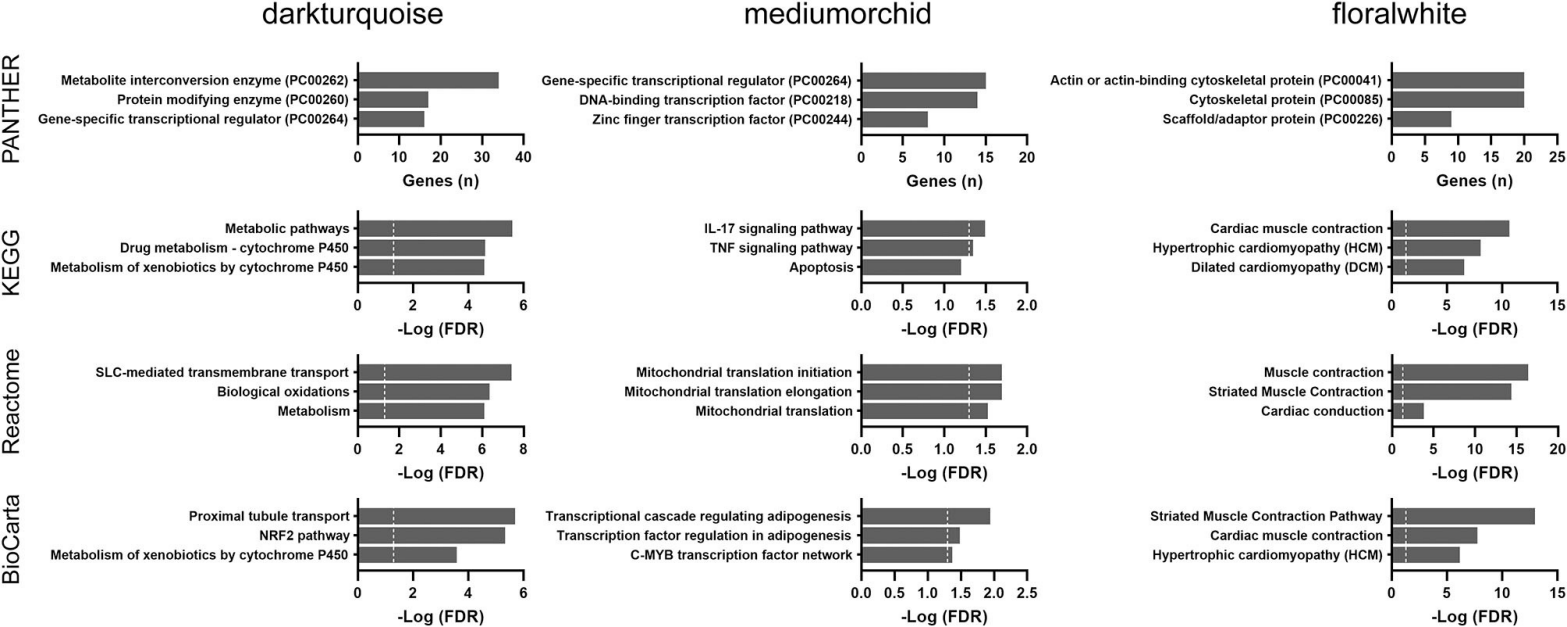
Over-representation pathway analysis of three top-ranked WGCNA modules in the TCGA THCA dataset: PANTHER protein class, KEGG, Reactome, and BioCarta. The top three classes or pathways are listed. Values above the dashed line represent significant enrichment (5% level of significance). FDR, false discovery rate.

To corroborate our exploratory findings, a third analysis, the WGCNA approach, was conducted. WGCNA transforms gene expression data into a gene co-expression network without specifying an arbitrary number of clusters and provides insights into co-expression modules that might underlie a given biological process. Three modules (*darkturquoise, mediumorchid*, and *floralwhite*) were significantly correlated with age based on the results of the WGCNA method (Supplementary Table 3). The highly correlated *darkturquoise* module was enriched for cytochrome P450 metabolism, thus underlining the robustness of the association between aging and metabolism. Pathway analyses of the *mediumorchid* module showed various enrichments in transcriptional regulation, inflammatory responses (IL-17 and TNF signaling), adipogenesis regulation, and mitochondrial translation (Figure 5). The *floralwhite* module consisted of actin or other cytoskeletal proteins that were grouped into muscle contraction or cardiomyopathy pathways.

Taken together, common pathways enriched in both the GSEA and WGCNA analyses from the TCGA cohort included cytochrome P450 metabolism, fatty acid metabolism/adipogenesis, mitochondrial functions, cytoskeletal proteins, and inflammatory cytokine signaling.

### GSEA and WGCNA Approaches in the GTEx Cohort

**Figure 6:**
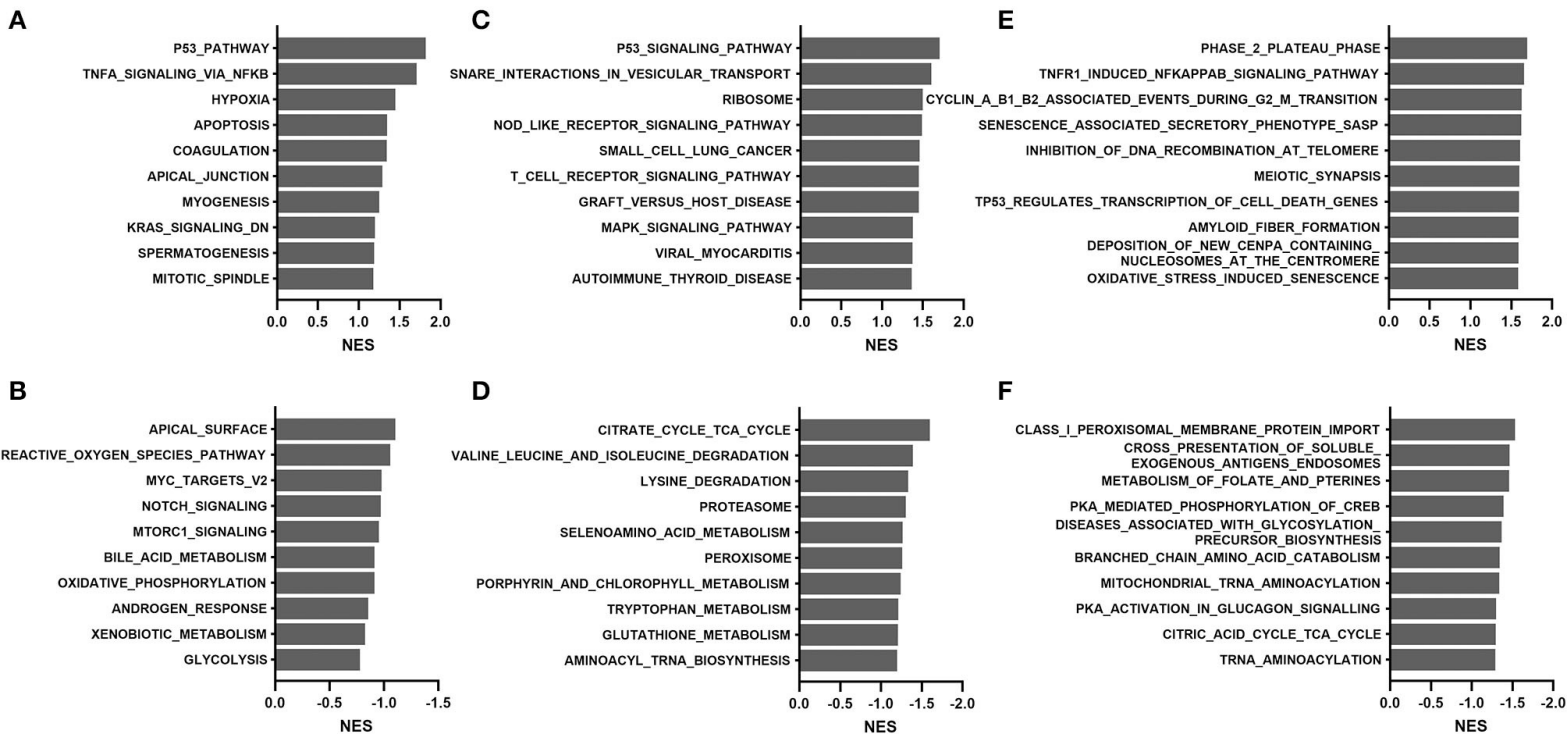
Gene set enrichment analysis of the GTEx dataset comparing the young (20–59 years) and old (60–79 years) groups: **(A,B)** hallmark, **(C,D)** KEGG, and **(E,F)** Reactome. **(A,C,E)**, positive enrichment; **(B,D,F)**, negative enrichment. The top 10 hallmarks or pathways are listed. NES, normalized enrichment score.

We further performed GSEA analyses to compare transcriptomes of the young (20–59 years) and old (60–79 years) groups in the GTEx cohort. As shown in Figure 6, positive enrichment pathways were associated with p53, apoptosis, immune system signaling, and senescence. In contrast, amino acid degradation, proteasomal activity, and mitochondria-related functions (bile acid metabolism, oxidative phosphorylation, and citric acid cycle) were negatively enriched. Proteasomal changes were not identified in the TCGA cohort but were previously reported via the WGCNA approach in the GTEx cohort (5).

**Figure 7:**
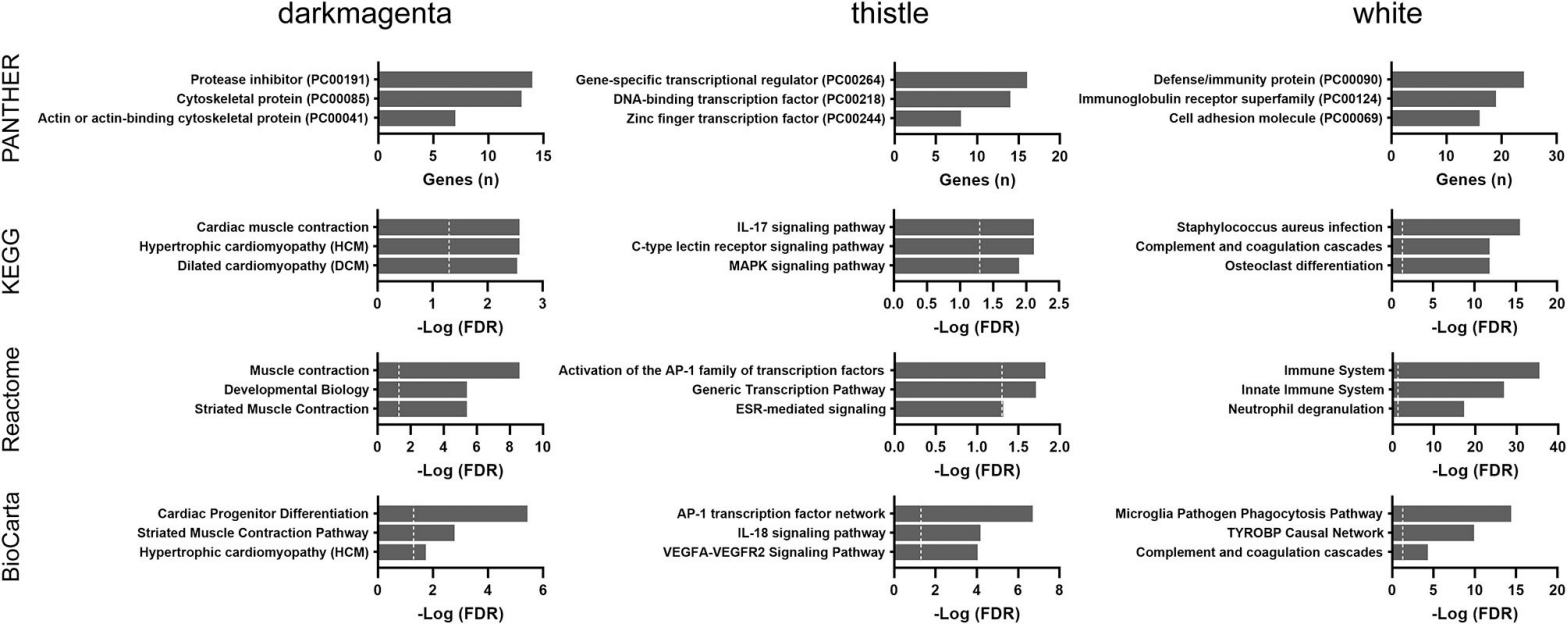
Over-representation pathway analysis of three top-ranked WGCNA modules in the GTEx dataset: PANTHER protein class, KEGG, Reactome, and BioCarta. The top three classes or pathways are listed. Values above the dashed line represent significant enrichment (5% level of significance). FDR, false discovery rate.

The updated GTEx database (v8 release) has almost double the number of samples than a previous analysis using the v6 release (5). We applied the WGCNA approach using the updated GTEx database and identified four modules that were significantly correlated with age groups (Supplementary Table 4). Pathway enrichment analyses were performed for the top three significant modules (*darkmagenta, thistle*, and *white*). As shown in Figure 7, the *darkmagenta* module contained genes involved in pathways very similar to that of the *floralwhite* module of the TCGA analysis, mostly actin or other cytoskeletal proteins. The *thistle* module had genes primarily associated with transcriptional regulation, consistent with the *mediumorchid* module of the TCGA analysis. The *white* module contained genes that participate in the immune/inflammation response, consistent with the findings of over-representation and GSEA analyses.

Collectively, immune and inflammatory responses were the most consistent age-associated changes across both the GSEA and WGCNA analyses in the GTEx cohort.

### Thyroid Differentiation

None of the 16 thyroid differentiation genes showed a significant correlation between their expression levels and age among the TCGA subjects. There was no significant correlation between the calculated thyroid differentiation index and patient age. For the GTEx cohort, three thyroid differentiation genes showed a negative correlation between age and expression: FOXE1 (*p* < 0.001), TPO (*p* = 0.007), and DUOX2 (*p* = 0.016).

## Discussion

Histological changes of the thyroid with advancing age include increased interfollicular fibrosis, reduction in follicle size, degeneration and flattening of epithelial cells, and various degrees of lymphocytic infiltration (13). The gene expression signature of aging in the thyroid gland is much less studied. A prior study identified three age-associated genes in the thyroid: PTCHD4, SP7, and ZMAT3 (4). Our results confirmed that PTCHD4 expression was positively correlated with age. Nonetheless, we did not detect an association between age and SP7 or ZMAT3 expression. The discrepancy likely results from different sample sources and different analytic strategies. Furthermore, the relatively small sample size of the TCGA cohort may restrict the generalizability of our findings to larger populations.

The prevalence of both hyperthyroidism and hypothyroidism rises with increasing age (14). In elderly patients, higher free thyroxine levels were associated with an increased risk of coronary artery disease (15). Progression from subclinical to overt thyroid dysfunction is more pronounced in patients who are positive for anti-thyroid antibodies. The large National Health and Nutrition Examination Survey (NHANES) study reported an age-dependent increase in the prevalence of anti-thyroid antibodies (16). Although a slight decrease in thyroid function leads to higher TSH levels in the elderly, paradoxically, the incidence of mild hyperthyroidism also increases with increasing age, especially in populations with iodine deficiency (17).

A major finding of our study is that immune and inflammatory responses were significantly associated with aging. Consistent with a previous report (5), our GTEx analysis suggested an upregulation in immune responses associated with aging, such as the T-cell receptor signaling pathway. In contrast, the GSEA procedure for the TCGA cohort showed an overall negative enrichment of the immune response. It should be noted that all GTEx samples with thyroiditis were excluded from the analysis. However, the status of thyroiditis in normal-appearing TCGA samples was unknown. When immune infiltrates increase in the thyroid parenchyma, the fraction of follicular epithelial cells decreases, thus interfering with the biological interpretation of the results of this study. Autoimmune thyroid disease may result from an imbalance in immune regulation. We previously demonstrated that natural killer cell-mediated cytotoxicity declined with aging in papillary thyroid cancer (18). Aging processes may have different activating and suppressive effects on diverse immune cell lineages.

We observed alterations in cytoskeletal proteins and possibly extracellular matrix organization with aging in the thyroid. Changes in the cytoskeleton were, nonetheless, less prominent in the analyses of the GTEx cohort, except for the *darkmagenta* module of the WGCNA approach. Several aging-correlated proteins that play a role in tissue homeostasis have been reported previously. Matrix metallopeptidase 28 (MMP28) expression was increased in the left ventricle of old mice compared to young controls (19). Additionally, the expression of microfibril associated protein 5 (MFAP5) in the trabecular bone was higher in the older group (20). These alterations may correspond to a typically subtle degree of diffuse fibrous interstitial expansion seen in the thyroid gland with advancing age.

An interesting finding of our pathway analysis is that amino acid metabolism, particularly tyrosine metabolism, was significantly associated with aging. Thyroid hormone biosynthesis involves the iodination of tyrosine in the thyroglobulin to form 3-iodotyrosine (MIT) and 3,5-diiodotyrosine (DIT). Therefore, alterations in tyrosine metabolism may affect thyroid hormone synthesis and the levels of tyrosine-derived metabolites and transmitters in the thyroid tissue. Interestingly, a recent study demonstrated that the levels of enzymes in the tyrosine degradation pathway increased with age in *Drosophila melanogaster*, and the downregulation of tyrosine degradation pathway significantly extended Drosophila lifespan (21).

The Drosophila study suggested that mitochondrial dysfunction serves as a signal to increase the expression of enzymes in the tyrosine degradation pathway (21). Among various aging theories, mitochondrial dysfunction has been observed in multiple organs and tissues (5, 22). Several metabolic pathways closely related to mitochondrial function were remarkably enriched in this study, including oxidative phosphorylation, respiratory electron transport, reactive oxygen species pathway, pyruvate and fatty acid metabolism, glycolysis, and Krebs cycle. It should be noted that the mitochondrion is a central regulator of apoptosis and senescence. Enriched apoptosis and p53 pathways seen in some analyses of the present study may also be linked to mitochondrial changes.

The previous study using the GTEx data reported an age-related deterioration in the thyroid differentiation score in women (5). Nonetheless, another analysis generated from the GTEx consortium is in agreement with the present observation that there was no association between age and the expression of 16 thyroid differentiation genes (4). In our opinion, it is unlikely that thyroid differentiation status plays an important role in thyroid dysfunction in the elderly. We believe that alterations in tyrosine metabolism and/or autoimmune thyroid disease remain the prevalent etiology of thyroid dysfunction in areas of iodine sufficiency (23).

We reproduced the results described in the previous GTEx study indicating that aging was associated with downregulation of genes related to proteasomal function (5). In our TCGA analysis, there was no association between age and expression levels of genes encoding proteasomes. Noteworthily, TCGA samples were collected during surgery for papillary thyroid cancer, while GTEx samples were obtained from postmortem donors. It seems that death classification based on the Hardy Scale (violent and fast death lasting <10 min, a fast death by natural causes lasting 10 min to 1 h, an intermediate rate of death lasting 1 to 24 h, and a slow death with a terminal phase lasting >1 day) was not adjusted in the previous GTEx analysis (5). More studies are needed to clarify whether pathways related to the proteasome and ubiquitin-mediated proteolysis are truly associated with aging in the thyroid gland.

We acknowledge a limitation of the current study that the TCGA tissue source was from patients with papillary thyroid cancer. Although TCGA transcriptome profiles were obtained from normal-appearing thyroid tissue, we could not exclude the possibility that the gene expression signature is influenced by field cancerization. As such, a cancer-primed neighborhood would have no gross change in normal follicular architecture (24, 25). The aging-associated upregulation of fatty acid metabolism seen in this study may contribute to dysregulation of lipid metabolism in thyroid cancer (26). A recent single-cell RNA sequencing study confirmed a premalignant state, in which cancer-primed cells with aberrant expression programs have yet to undergo evident changes in cellular morphology (27). Hence, some caution in interpreting the results is warranted.

**Figure 8:**
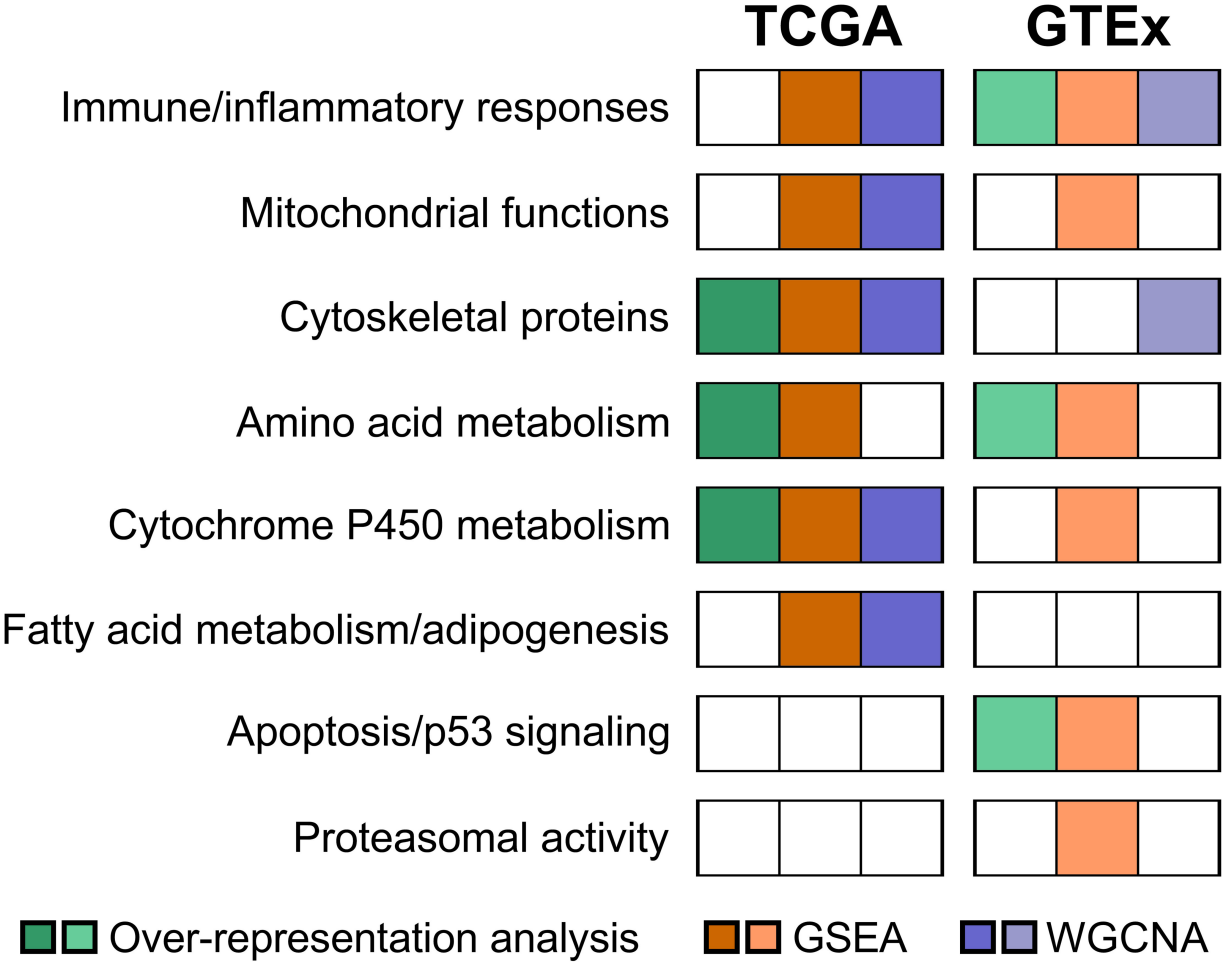
A summary chart showing pathways associated with aging in the thyroid gland.

In conclusion, the present study reveals that thyroid aging is associated with alterations in immune and inflammatory responses, mitochondrial function, cytoskeletal proteins, as well as amino acid and cytochrome P450 metabolism (Figure 8). Thyroid differentiation appears to be largely unaffected. Continued exploration of transcriptomic characteristics associated with aging may provide new insight into thyroid disorders in the geriatric population.

## Data Availability Statement

The original contributions presented in the study are included in the article/Supplementary Material, further inquiries can be directed to the corresponding author/s.

## Ethics Statement

Ethical review and approval was not required for the study on human participants in accordance with the local legislation and institutional requirements. Written informed consent for participation was not required for this study in accordance with the national legislation and the institutional requirements.

## Author Contributions

C-LL and S-PC: conceptualization and writing–original draft. C-LL, M-NC, and S-PC: data curation. C-LL, Y-CH, and S-PC: formal analysis. M-NC and Y-CH: writing–review and editing. All authors have read and approved the final manuscript.

## Funding

This work was supported by research grants from the Ministry of Science and Technology of Taiwan (MOST-109-2314-B-195-018-MY3) and MacKay Memorial Hospital (MMH-E-111-08 and MMH-11112).

## Conflict of Interest

The authors declare that the research was conducted in the absence of any commercial or financial relationships that could be construed as a potential conflict of interest.

## Publisher's Note

All claims expressed in this article are solely those of the authors and do not necessarily represent those of their affiliated organizations, or those of the publisher, the editors and the reviewers. Any product that may be evaluated in this article, or claim that may be made by its manufacturer, is not guaranteed or endorsed by the publisher.
